# Identification of novel gene and pathway targets for human epilepsy treatment

**DOI:** 10.1186/s40659-015-0060-5

**Published:** 2016-01-07

**Authors:** Ying Jin, Chunzhe Zhao, Lihui Chen, Xiangyu Liu, Shuxiao Pan, Dongsheng Ju, Jing Ma, Jinying Li, Bo Wei

**Affiliations:** Department of Neurology, Jilin Oilfield General Hospital, Songyuan, 131200 China; The Second Division of Neurosurgery, Departments of Neurosurgery, The China-Japan Union Hospital of Jilin University, 126 Xiantai Street, Changchun, 130033 Jilin Province China

**Keywords:** Epilepsy, Differentially expressed genes, Functional annotation, Protein–protein interaction network, Co-expression module

## Abstract

**Background:**

The aim of this study was to explore epilepsy-related mechanism so as to figure out the possible targets for epilepsy treatment.

**Methods:**

The gene expression profile dataset GES32534 was downloaded from Gene Expression Omnibus database. We identified the differentially expressed genes (DEGs) by Affy package. Then the DEGs were used to perform gene ontology (GO) and pathway enrichment analyses. Furthermore, a protein–protein interaction (PPI) network was constructed with the DEGs followed by co-expression modules construction and analysis.

**Results:**

Total 420 DEGs were screened out, including 214 up-regulated and 206 down-regulated genes. Functional enrichment analysis revealed that down-regulated genes were mainly involved in the process of immunity regulation and biological repairing process while up-regulated genes were closely related to transporter activity. PPI network analysis showed the top ten genes with high degrees were all down-regulated, among which *FN1* had the highest degree. The up-regulated and down-regulated DEGs in the PPI network generated two obvious sub-co-expression modules, respectively. In up-co-expression module, *SCN3B* (sodium channel, voltage gated, type III beta subunit) was enriched in GO:0006814 ~ sodium ion transport. In down-co-expression module, *C1QB* (complement C1s), *C1S* (complement component 1, S subcomponent) and *CFI* (complement factor I) were enriched in GO:0006955 ~ immune response.

**Conclusion:**

The immune response and complement system play a major role in the pathogenesis of epilepsy. Additionally, *C1QB*, *C1S*, *CFI*, *SCN3B* and *FN1* may be potential therapeutic targets for epilepsy.

## Background

Epilepsy characterized by epileptic seizures, is a kind of neurological disorder [1]. Epileptic seizure is defined as “a state produced by an abnormal excessive neuronal discharge within the central nervous system” in terms of the mechanism. Seizure may originate from any part of the brain and spread to other areas [2]. It has been reported that there are approximately one million people diagnosed with seizure or epilepsy in an emergency department annually in the United States [3]. Patients with epilepsy or seizure often present decreased level of consciousness and disorder to response to environment. Therefore, better understanding of the mechanisms underlying alteration of consciousness in epilepsy is important for the treatment of patients with epilepsy [4].

Currently, the pathogenesis of epilepsy has been widely investigated. Epilepsy may develop from the up-regulated excitatory circuits or down-regulated excitatory due to brain injury [5]. A previous study indicated that a loss of glutamine synthetase was the direct factor for high level of extracellular glutamate that played a key role in the occurrence of epileptic seizures [6]. Turrin et al. [7] suggested a different mechanism related with pro-inflammatory expression in regulating the innate immune reaction in response to seizures, which gave a new perspective for epilepsy related neuropathology. Furthermore, it is reported that specific inflammatory pathways are chronically activated during epileptogenesis and persist in chronic epileptic tissue, suggesting they may contribute to the etiopathogenesis of temporal lobe epilepsy [8]. However, current studies for genetic mechanism underlying epilepsy has not been clearly defined. Besides, there are not enough epilepsy related candidate genes which can be used as treatment targets.

In the present study, we investigated the global gene expression profile GSE32534, which was supplied by Niesen et al. [9]. Additionally, He et al. [10] used this expression profile to perform gene set enrichment analysis and identified several epilepsy related transcription factors and pathways. However, they did not analyze the protein–protein interaction (PPI) network and co-expression modules associated with epilepsy. PPI and co-expression modules analyses can provide new insights into protein function, besides, they may help to uncover the generic organization principles of functional cellular networks [11]. Therefore, based on the dataset of GSE32534, we firstly identified the differentially expressed genes (DEGs) between healthy and epilepsy specimens. Then, gene ontology (GO) function and Kyoto encyclopedia of genes and genomes (KEGG) pathway analyses were performed on the selected DEGs to explore the biological roles of these DEGs. Finally, we constructed and analyzed PPI network and co-expression modules. We aimed to further explore the molecular mechanism underlying epilepsy and explore novel gene targets for epilepsy therapy.

## Methods

### Data pre-processing and differentially expressed gene analysis

The epilepsy related dataset (accession number: GSE32534) that was contributed by Niesen et al. [9] was downloaded from the Gene Expression Omnibus (GEO, http://www.ncbi.nlm.nih.gov/geo/) based on Affymetrix Rat Genome U34 Array (Affymetrix Inc., Santa Clara, CA, USA). Data from ten peritumoral cortex tissue specimens were available for further analysis, including five samples from epilepsy and five from non-epilepsy patients with low grade brain tumor.

The probe-level data were converted into expression matrix by RMA (function in Affy package) in R language. Probe numbers were converted into gene names using Bioconductor annotation function in R language combined with annotation information in microarray platform GPL570 (HG-U133_Plus_2). The expression values of multiple probes for a given gene were reduced to a single value by taking the average expression value. After the expression data were normalized [12], the DEGs (epilepsy vs. non-epilepsy) were identified by Limma package [13] in R language. Genes with |log_2_FC (fold change)| > 1 and p < 0.05 were defined as DEGs.

### Functional enrichment analysis

Database for annotation, visualization, and integrated discovery (DAVID, http://david.abcc.ncifcrf.gov/) [14, 15], an online tool, consists of an integrated biological knowledgebase and analytic tools aimed at systematically extracting biological meaning from large gene or protein lists [16]. In this study, the GO functional and KEGG pathway enrichment analyses for the screened DEGs was performed by DAVID. The multiple testing correction was performed using Benjamini–Hochberg (HB) method [17], and fold discovery rate (FDR) <0.05 was chosen as the cut-off criterion.

### Construction of protein–protein interaction network

Search tool for the retrieval of interacting genes/proteins (STRING, http://string-db.org/) [18] was used to construct protein–protein interaction network among DEGs. Genes with correlation coefficient higher than 0.7 were mapped to PPI network, which was visualized by Cytoscape (http://www.cytoscape.org/) [19]. Igraph package in R language was used to calculate the connectivity degree of each node.

### Construction of co-expression module

Expression similarity coefficient was calculated to select co-expression genes among DEGs. The co-expression module was constructed by the DEGs with expression similarity coefficient >0.99 and p < 0.001. GO and KEGG pathway enrichment analyses were performed for modular genes using the DAVID online tool.

## Results

### DEGs identification

**Fig. 1:**
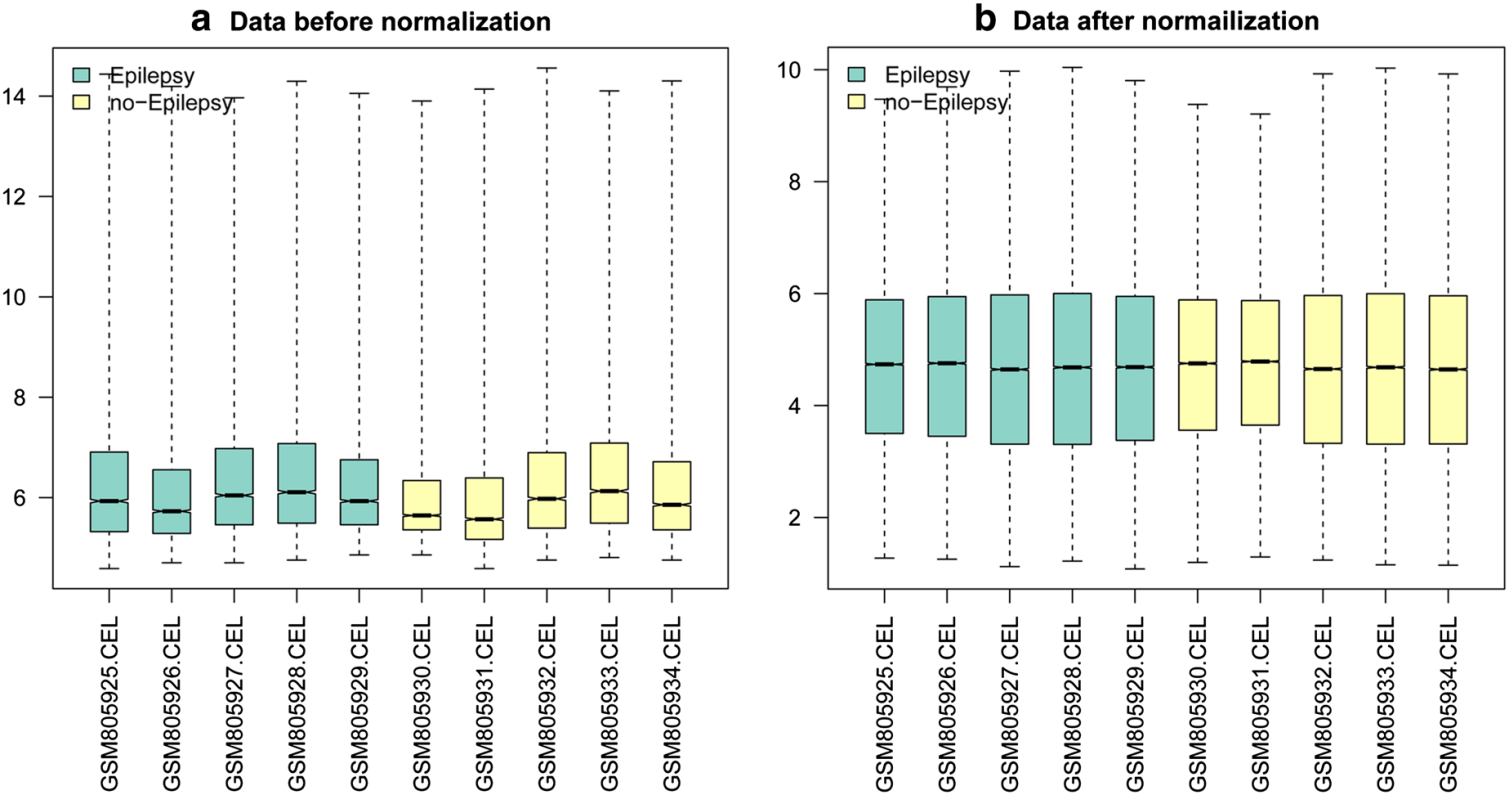
Box plot of expression data before and after normalization. The horizontal axis stands for different samples, while the vertical axis represents expression value. The *black* *line* in the box represents the expression median for each sample. **a** Data before normalization. **b** Data after normalization. After preprocessing, the *black* *lines* of box plot are almost on the same *straight line*, indicating a high level of normalization

**Fig. 2:**
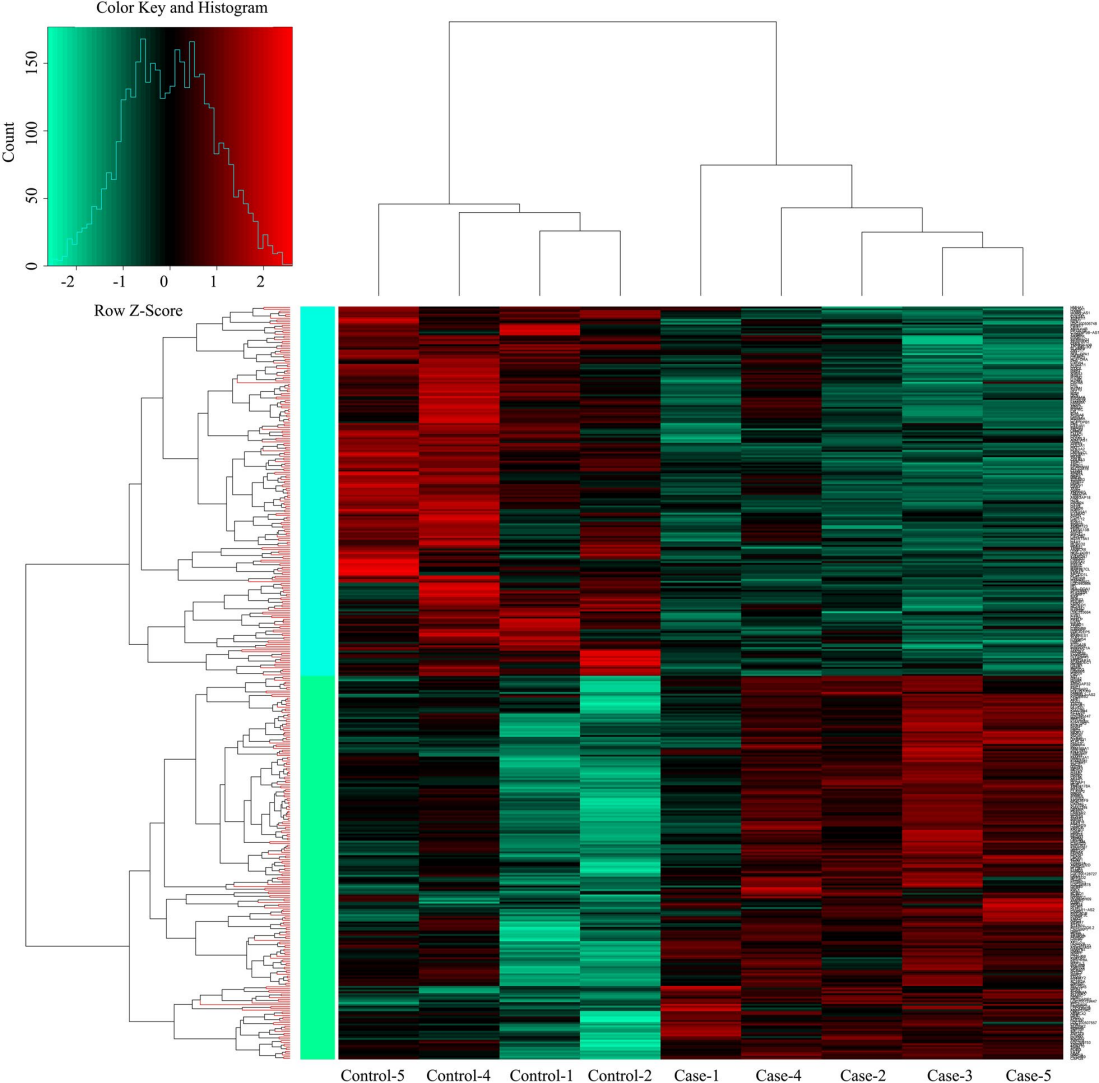
Cluster analysis of the differentially expressed genes. Each *row* represents a single gene and each *column* a single sample. The data are colored *red* for high abundance/up-regulation and *green* for low abundance/down-regulation

After preprocessing, we obtained the expression values of 19,944 genes. The normalization showed a good result on a wide variety of array (Fig. 1). Total 420 DEGs were screened out, including 214 up-regulated and 206 down-regulated genes. Hierarchical clustering indicated that the DEGs could clearly distinguish epilepsy samples from the normal controls (Fig. 2).

## GO functional and KEGG pathway enrichment analyses

The results of GO and KEGG enrichment analyses were displayed in Table 1. The significantly enriched GO terms of up-regulated DEGs were closely associated with cell–cell signaling, synaptic transmission and transmission of nerve impulse. The down-regulated DEGs were significantly enriched in immunity regulation and repairing process of injured tissues. The significant pathway that enriched by up- and down-regulated DEGs was hsa04720:Long-term potentiation and hsa04510:Focal adhesion, respectively.

**Table 1:** GO and KEGG pathway enrichment analysis for identified DEGs

Category	Term	Count	p value
*Up-regulated genes*			
GOTERM_BP_FAT	GO:0050877 ~ neurological system process	26	2.15E−04
GOTERM_BP_FAT	GO:0019226 ~ transmission of nerve impulse	22	2.01E−11
GOTERM_BP_FAT	GO:0006811 ~ ion transport	21	4.75E−05
GOTERM_BP_FAT	GO:0007267 ~ cell–cell signaling	20	4.97E−06
GOTERM_BP_FAT	GO:0007268 ~ synaptic transmission	19	5.63E−10
GOTERM_CC_FAT	GO:0031224 ~ intrinsic to membrane	70	0.007687949
GOTERM_CC_FAT	GO:0016021 ~ integral to membrane	65	0.030340215
GOTERM_CC_FAT	GO:0005886 ~ plasma membrane	63	4.81E−06
GOTERM_CC_FAT	GO:0044459 ~ plasma membrane part	48	9.49E−08
GOTERM_CC_FAT	GO:0005887 ~ integral to plasma membrane	23	0.00337979
GOTERM_MF_FAT	GO:0005216 ~ ion channel activity	15	1.16E−05
GOTERM_MF_FAT	GO:0022838 ~ substrate specific channel activity	15	1.63E−05
GOTERM_MF_FAT	GO:0015267 ~ channel activity	15	2.40E−05
GOTERM_MF_FAT	GO:0022803 ~ passive transmembrane transporter activity	15	2.46E−05
GOTERM_MF_FAT	GO:0022836 ~ gated channel activity	13	2.69E−05
KEGG_PATHWAY	hsa04720:Long-term potentiation	7	1.89E−05
KEGG_PATHWAY	hsa05014:Amyotrophic lateral sclerosis (ALS)	5	9.61E−04
KEGG_PATHWAY	hsa04020:Calcium signaling pathway	7	0.003316868
KEGG_PATHWAY	hsa04080:Neuroactive ligand-receptor interaction	8	0.005123351
KEGG_PATHWAY	hsa04310:Wnt signaling pathway	5	0.037620947
*Down-regulated genes*			
GOTERM_BP_FAT	GO:0006955 ~ immune response	40	1.23E−16
GOTERM_BP_FAT	GO:0006952 ~ defense response	30	1.65E−10
GOTERM_BP_FAT	GO:0009611 ~ response to wounding	26	3.53E−09
GOTERM_BP_FAT	GO:0007155 ~ cell adhesion	26	7.74E−07
GOTERM_BP_FAT	GO:0022610 ~ biological adhesion	26	7.94E−07
GOTERM_MF_FAT	GO:0005198 ~ structural molecule activity	20	4.49E−05
GOTERM_MF_FAT	GO:0005509 ~ calcium ion binding	18	0.018905046
GOTERM_MF_FAT	GO:0005201 ~ extracellular matrix structural constituent	13	1.09E−10
GOTERM_MF_FAT	GO:0042802 ~ identical protein binding	13	0.041784269
GOTERM_MF_FAT	GO:0004857 ~ enzyme inhibitor activity	12	1.53E−04
GOTERM_CC_FAT	GO:0005576 ~ extracellular region	70	4.18E−15
GOTERM_CC_FAT	GO:0005886 ~ plasma membrane	69	0.002166744
GOTERM_CC_FAT	GO:0044421 ~ extracellular region part	50	4.52E−17
GOTERM_CC_FAT	GO:0005615 ~ extracellular space	30	2.56E−08
GOTERM_CC_FAT	GO:0005578 ~ proteinaceous extracellular matrix	28	1.34E−14
KEGG_PATHWAY	hsa04510:Focal adhesion	16	4.33E−07
KEGG_PATHWAY	hsa05322:Systemic lupus erythematosus	14	3.27E−09
KEGG_PATHWAY	hsa04610:Complement and coagulation cascades	13	4.92E−10
KEGG_PATHWAY	hsa04512:ECM-receptor interaction	13	5.21E−09
KEGG_PATHWAY	hsa04060:Cytokine–cytokine receptor interaction	10	0.02253563

Count represents the gene number

## PPI network analysis

**Fig. 3:**
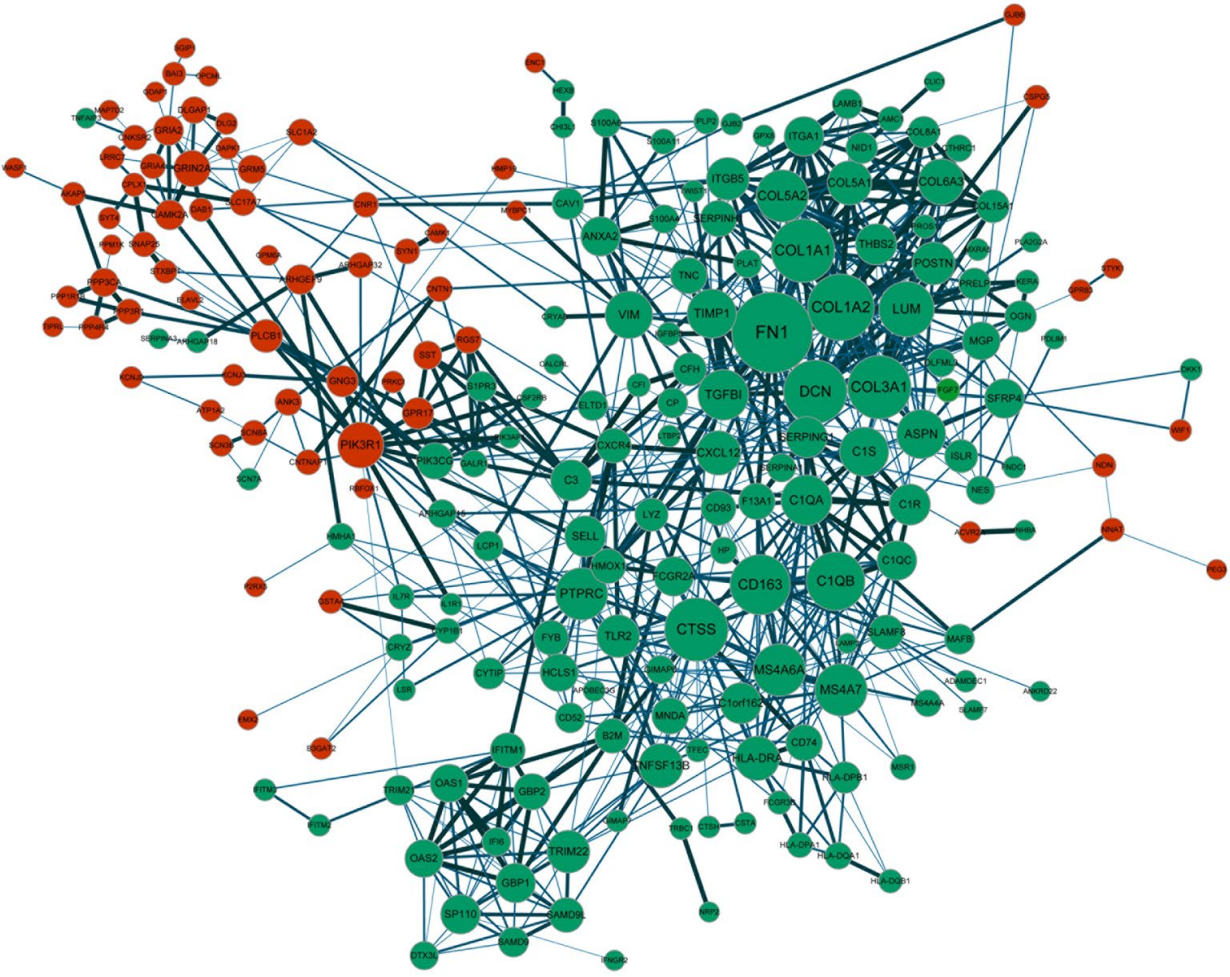
PPI network. *Green* *nodes* represent down-regulated genes while *red* *nodes* represent up-regulated genes. The size of the *nodes* is positively correlated with the degrees of genes. The *thickness of line* is determined by the correlation coefficient provided by STRING

In the PPI network, rare interactions were observed between the up- and the down-regulated genes, while most interactions were observed within the up-regulated genes or the down-regulated genes (Fig. 3). Besides, nodes degree was analyzed for genes in PPI network to obtain the information of hub nodes. Ten DEGs were considered as hub genes, such as *FN1* (fibronectin 1), *COL1A2* (collagen alpha-2), and *C1QB* (complement C1s) (Table 2).

**Table 2:** Top genes with high node degrees in PPI network

Node	Degree	log_2_FC
FN1	39	−1.024861275
COL1A2	30	−2.064034307
DCN	28	−1.628988128
CTSS	28	−1.089500003
COL3A1	28	−2.33411558
COL1A1	28	−2.131930656
CD163	26	−1.470072732
C1QB	25	−1.864805026
LUM	23	−2.114081932
PTPRC	21	−1.083176333

## Co-expression modules

**Fig. 4:**
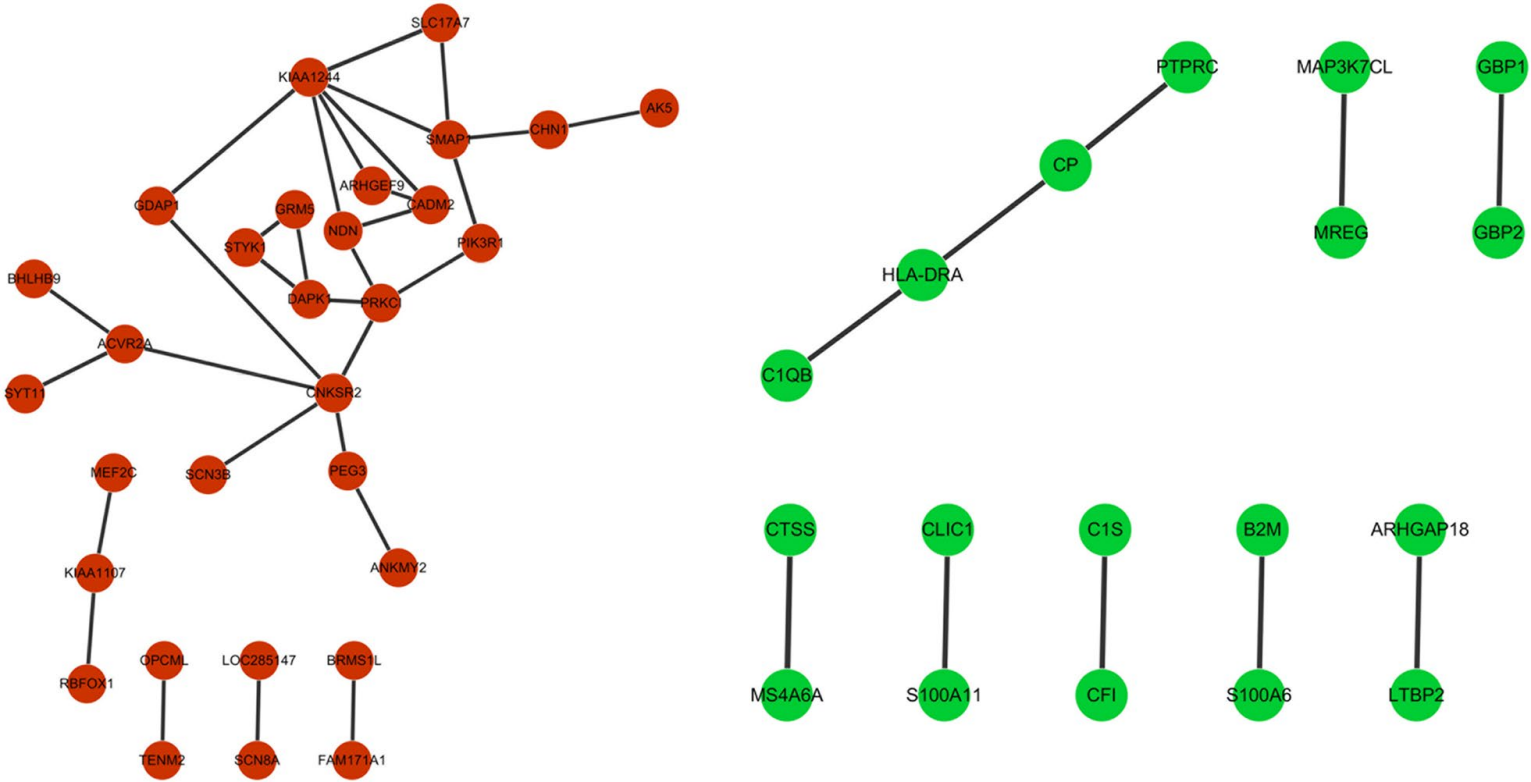
Co-expression module. *Green* *nodes* represent down-regulated genes while *red* *nodes* represent up-regulated genes

The co-expression modules were constructed with up- and down-regulated genes, respectively (Fig. 4). As shown in Table 3, obviously enriched GO terms of genes in up-co-expression module were GO:0006814 ~ sodium ion transport, GO:0030695 ~ GTPase regulator activity and GO:0060589 ~ nucleoside-triphosphatase regulator activity. The down-regulated genes in the co-expression modules were mainly associated with immunity regulation and cell damage repairing processes. For instance, *SCN3B* (sodium channel, voltage gated, type III beta subunit) was enriched in GO:0006814 ~ sodium ion transport. *C1QB*, *C1S* (complement component 1, S subcomponent), and *CFI* (complement factor I) were enriched in GO:0006955 ~ immune response and GO:0050778 ~ positive regulation of immune response.

**Table 3:** GO functional and KEGG pathway enrichment analysis on genes in co-expression modules

ID	Term	Description	Count	p value
Up-co-expression	GOTERM_BP_FAT	GO:0016310 ~ phosphorylation	5	0.023081277
	GOTERM_BP_FAT	GO:0006793 ~ phosphorus metabolic process	5	0.043264378
	GOTERM_BP_FAT	GO:0006796 ~ phosphate metabolic process	5	0.043264378
	GOTERM_BP_FAT	GO:0006915 ~ apoptosis	4	0.049975204
	GOTERM_BP_FAT	GO:0006814 ~ sodium ion transport	3	0.014081453
	GOTERM_CC_FAT	GO:0005886 ~ plasma membrane	11	0.02927495
	GOTERM_CC_FAT	GO:0044459 ~ plasma membrane part	8	0.033090626
	GOTERM_MF_FAT	GO:0030695 ~ GTPase regulator activity	4	0.020009788
	GOTERM_MF_FAT	GO:0060589 ~ nucleoside-triphosphatase regulator activity	4	0.021203692
	GOTERM_MF_FAT	GO:0008289 ~ lipid binding	4	0.02652355
	GOTERM_MF_FAT	GO:0031402 ~ sodium ion binding	3	0.012457524
	GOTERM_MF_FAT	GO:0005543 ~ phospholipid binding	3	0.027145214
Down-co-expression	GOTERM_BP_FAT	GO:0006955 ~ immune response	9	1.01E−07
	GOTERM_BP_FAT	GO:0050778 ~ positive regulation of immune response	6	2.45E−07
	GOTERM_BP_FAT	GO:0048584 ~ positive regulation of response to stimulus	6	2.73E−06
	GOTERM_BP_FAT	GO:0002684 ~ positive regulation of immune system process	6	2.84E−06
	GOTERM_BP_FAT	GO:0006952 ~ defense response	6	2.72E−04
	GOTERM_MF_FAT	GO:0048154 ~ S100 beta binding	2	0.003999687
	GOTERM_MF_FAT	GO:0048306 ~ calcium-dependent protein binding	2	0.031586688
	GOTERM_MF_FAT	GO:0042803 ~ protein homodimerization activity	3	0.042687029
	GOTERM_MF_FAT	GO:0004175 ~ endopeptidase activity	3	0.052602322
	GOTERM_MF_FAT	GO:0005509 ~ calcium ion binding	4	0.05926993
	GOTERM_CC_FAT	GO:0005576 ~ extracellular region	7	0.020898219
	KEGG_PATHWAY	hsa04610:Complement and coagulation cascades	3	0.003647135
	KEGG_PATHWAY	hsa04612:Antigen processing and presentation	3	0.005241774
	KEGG_PATHWAY	hsa05322:Systemic lupus erythematosus	3	0.007393725

Count represents the gene number

## Discussion

Epilepsy is defined as a chronic brain disorder and its genetic mechanism has not been elucidated clearly, which hampered the development of novel preventive and treatment management. In this study, we explored the molecular mechanism of human epilepsy by using bioinformatics methods and attempted to give a new insight to the design of good therapy. Total 214 up-regulated genes and 206 down-regulated DEGs between epilepsy and control group were screened out. According to PPI network analysis, top ten DEGs with high node degrees were all down-regulated. Among them, *FN1* showed the highest degree, which was closely associated with cell transport activity.

The data of GO analysis showed that the down-regulated DEGs in epilepsy patients were significantly enriched in immune response and defense response related biological processes. Recent evidence has suggested that the innate and adaptive immune reaction is involved in the epilepsy, which was implicated in the inflammatory processes within the brain [20]. It has been proved that the immune system and the nervous system maintain extensive communication [21]. The inflammation of brain contributed to the local neuronal excitability that resulted in seizures [22]. Importantly, several inflammatory mediators, including temporal lobe epilepsy and cortical dysplasia-related epilepsy, have been detected in surgically resected brain tissue from epilepsy patients [23]. Our results further supported that immune response and inflammatory processes might constitute a crucial mechanism in the pathophysiology of epilepsy.

Specially, in our study, several DEGs were found significantly enriched in immune regulation related terms in the co-expression module, which was consistent with the results of GO functional analysis. These DEGs included *C1QB*, *C1S*, *CFI*, *PTPRC*, *HLA*-*DRA* and *B2M*. Previous study has found that the complement system represents an essential effector of both humoral immunity and cellular immunity [24]. Among these DEGs in the co-expression module, *C1QB* and *C1S* encoded the subunits of human complement component C1 and play roles in the complement activation pathway. C1 deficiency and impaired activation of the complement classical pathway generally leads to severe immune complex disease [25]. *C1QB* has been found to be a epilepsy related gene and the expression of *C1QB* mRNA declines in brain peritumoral tissues of patients with tumor-induced epilepsy [26]. Besides, expression of various complement components, such as C1q, C3c, and C3d, has been observed in reactive astrocytes within the sclerotic hippocampus of people with temporal lobe epilepsy [27]. Interestingly, protein encoded by *CFI* is essential for regulating the complement cascade. *CFI* can inhibit the activation of complements by the inactivation of C3b and C4b and the normal combination of C3 and C5. The persistence of complement activation could contribute to a sustained inflammatory response and could destabilize neuronal networks [24]. Thus, the down-regulation of *CFI* may promote the activation of complements to accelerate the progression of epilepsy. Importantly, *CFI* has significant interconnectivity with *C1S* in this paper. Besides, *CFI*, *C1S* and *C1QB* jointly enriched in the complement activation pathway according to the results of GO and pathway enrichment analysis. Taken together, we proposed that complement system might play key roles in the development of epilepsy by dysregulating the expression of *C1QB*, *C1S* and *CFI*.

In addition to down-co-expression module, in the up-co-expression module, *SCN3B* was found enriched in GO terms related to sodium ion transport. The rapid and selective transport of sodium ion through sodium channels is essential for initiating action potentials within excitable cells [28]. Study has found that changes in the function of many ion channels, including sodium channels, contribute to the epileptogenesis [29]. Baek et al. [30] reported that voltage-gated sodium channels could initiate action potentials in brain neurons, and mutation of the channels was associated with several forms of inherited epilepsy. Importantly, *SCN3B*, an enriched DEGs of this term, is demonstrated to encode voltage-gated sodium channels. In adult brain, there are four accessory β subunits, including *SCN1B*, *SCN2B*, *SCN3B* and *SCN4B*. The β subunits are thought to affect trafficking and gating of the voltage-gated sodium channels [31]. Therefore, we speculated that *SCN3B* might play an important role in the progression of epilepsy through sodium channels.

Furthermore, in our study, *FN1* was found to have the highest degree in PPI network. *FN1* is a multifunctional, extracellular matrix glycoprotein which contains several distinct domains that may bind to cell surfaces. Fibronectin has been reported to play a role in cellular morphology, phagocytosis, and wound repair [32]. Moreover, fibronectin was reported to be involved in astroglial proliferation. Previous study revealed that astrocytes play a major role in the regulation of the immune/inflammatory response in several human central nervous system diseases [33, 34]. In epilepsy-associated pathologies, astrocytes in tissues of patients with epilepsy undergo significant changes in their physiological properties [35]. As a result, we can predict that *FN1* may be a candidate molecular marker associated with the occurrence and progression of epilepsy patients by affecting the astrocytes.

In conclusion, our study provided a comprehensive bioinformatics analysis of DEGs and functions which may be involved in the progression of epilepsy. Immune response, complement system and sodium ion transport may play major roles in the pathogenesis of epilepsy. The dysregulation of *C1QB*, *C1S*, *CFI*, *SCN3B* and *FN1* may be used potential gene targets for epilepsy treatment. Although there are some novel findings, a lack of experimental evidences is a limitation in our study. Further experimental studies should be conducted to validate our findings in the future.

## Abbreviations

HB: Benjamini–Hochberg
*COL1A2*: collagen alpha-2
DAVID: database for annotation, visualization, and integrated discovery
DEGs: differentially expressed genes
FDR: fold discovery rate
*FN1*: fibronectin 1
KEGG: Kyoto encyclopedia of genes and genomes
GEO: gene expression omnibus
GO: gene ontology
STRING: search tool for the retrieval of interacting genes/proteins
